# Partitioning of soil phosphorus regulates competition between *Vaccinium vitis-idaea* and *Deschampsia cespitosa*

**DOI:** 10.1002/ece3.771

**Published:** 2013-10-01

**Authors:** Mohd F Ahmad-Ramli, Thomas Cornulier, David Johnson

**Affiliations:** Institute of Biological and Environmental Sciences, University of AberdeenCruickshank Building, St Machar Drive, Aberdeen, AB24 3UU, UK

**Keywords:** Competition, niche differentiation, organic phosphorus, peatlands, phosphomonoesterase, phytase, plant nutrition

## Abstract

It has been hypothesized that the wide range of forms and complexities of phosphorus (P) in soil may result in resource partitioning that contributes to the maintenance of plant species diversity. Here, we test whether the graminoid, *Deschampsia cespitosa*, and the ericaceous shrub, *Vaccinium vitis-idaea*, which often coexist, display preferences in utilization of P forms, and differ in their production of extracellular P-degrading enzymes. We provided plants with no additional P, or P forms with decreasing lability, namely sodium phosphate (SP), D-glucose 6 phosphate (DG6P), sodium phytate (PASS), and a combination of SP, DG6P, and PASS. We also tested if preferences for P forms affected the competitive outcomes between the two species compared between conspecifics, as indicated by shoot biomass and acquisition of nitrogen (N) and P. Both *D. cespitosa* and *V. vitis-idaea* produced the greatest biomass when supplied with a mix of all three forms of P. Of the three forms of P tested alone, shoot biomass produced by both species was least when supplied with SP. *D. cespitosa* performed better when grown with PASS or a mix of all P forms compared with the performance of *V. vitis-idaea* on these substrates. This was reflected by substantially greater phytase activity on the surface of its roots compared with *V. vitis-idaea*. In contrast, *V. vitis-idaea* produced more phosphomonoesterase to hydrolyze the simple organic P form, DG6P. Although N was kept constant in the treatments, the ability of plants to acquire it was dependent on species identity, competition, and P supply. These findings provide direct evidence for preferences toward specific forms of P and indicate a key role played by organic forms of P. The results support the idea that partitioning for soil P is one factor regulating plant competition, and ultimately, community composition. Our data also highlight the importance of the interplay between P supply and N acquisition.

## Introduction

The availability of phosphorus (P) is increasingly recognized as a key nutrient that limits productivity, either on its own or in combination with other mineral nutrients like N (Wassen et al. 2005). In most organic soils, P availability is often considered to be very low because of the acidity of many peat soils, which results in precipitation of highly insoluble Fe or Al phosphates, or occlusion of P in Fe or Al complexes. Between 30% and 65%, but sometimes >90%, of the total soil, P is found in organic forms (Harrison 1987) but this pool has traditionally been considered to be unavailable to plants.

The forms of P found in soils vary in structure and lability such that their recalcitrance often declines in the order inositol phosphates, diesters, simple monoesters, and orthophosphate (Turner et al. 2002a; Turner et al. 2003a,b). Thus, most soils contain a plethora of P forms, the heterogeneity of which gives rise for the potential for niche complementarity whereby plants may be more adept at utilizing specific P sources (Turner 2008). The conceptual model outlined by Turner (2008) is a development of ideas based on differential utilization and availability of N forms (McKane et al. 2002) but is so far untested in the context of P utilization. However, there is considerable circumstantial evidence underpinning the ideas described in the model. For example, evidence from laboratory microcosms containing the grass *Agrostis capillaris* shows that phytate is hydrolyzed and taken up by the plant within hours of exposure to the root systems and thus contributes significantly to its nutritional demands (Macklon et al. 1997).

Our understanding of the composition and turnover of organic P in soil has increased markedly, in many cases by application of nuclear magnetic resonance (NMR) imaging, and these advances demonstrate a rich diversity of P forms. For example, we now know that the relative proportions of organic forms of P often varies in soil (Turner et al. 2002b), and correlative-based approaches suggest that vegetation composition (Cross and Schlesinger 2001) may affect this.

Plants respond to P deficiency in a variety of ways, including changing root morphology, increasing production of extracellular phosphatases, upregulating P transporter genes, and forming symbioses with microorganisms, such as mycorrhizal fungi that have adaptations enabling them to acquire more P from soil. Plant roots have a similar capability as many soil microorganisms to secrete phosphatases, which release inorganic P by hydrolysis of ester bonds between organic carbon and P (Sahu et al. 2007). There is evidence that certain plants are capable of hydrolyzing organic P compounds in P deficient circumstances (Felipe et al. 1979; Tarafdar and Claassen 2003) but that this may differ according to the species of plant, thus supporting the idea of resource partitioning for P in soil (Turner 2008). For example, P-degrading enzymes vary considerably between species and functional type of plants (Johnson et al. 1999; Phoenix et al. 2004; Venterink 2011), and it has been suggested that this reflects different affinities for specific components of the heterogeneous soil organic P pool (Turner 2008). The production of phosphatases by *Eriophorum vaginatum* has been estimated to account for 69% of its annual P demand (Kroehler and Linkins 1988). *Plantago lanceolata* and *Rumex acetosella*, both of which are abundant in extensively grazed pastures, differ markedly in their utilization of soil P fractions (Fransson et al. 2003). Similarly, the formation of different types of mycorrhizas, or possibly colonization by different species of mycorrhizal fungi, may also promote partitioning of soil P. For example, ectomycorrhizal fungi are able to access phosphate esters and inositol phosphates (Antibus et al. 1992), while ericoid mycorrhizal fungi can efficiently use phosphate diesters (Leake and Miles 1996).

A further factor leading to P partitioning is the potential of plants to produce different types of P-degrading enzymes. Phosphomonoesterase is active under both alkaline and acid conditions (Criquet et al. 2004), and these enzymes differ in their reaction on different substrates. Phytase, also known as myo-insitolhexakis phosphate phosphohydrolase, is a phosphatase that hydrolyzes sodium phytate, releasing inorganic free orthophosphate (Wyss et al. 1999). Phytase can be secreted by plant roots (Li et al. 1997), especially when grown in P deficient conditions.

Despite the wide range of data available suggesting that partitioning of soil P has potential to occur, and the development of a theoretical model bringing these lines of evidence together (Turner 2008), there has been no empirical test of this hypothesis. This lack of evidence is a major gap in our understanding of what shapes plant community composition. It also limits our ability to determine how nutrient availability and acquisition may affect competition among individuals and species of plants. For example, the ability of two plants to utilize different forms of P in soil may alleviate competition and be a mechanism promoting coexistence.

Here, we grew two species of acid tolerant plants, *Vaccinium vitis-idaea* (a dwarf shrub) and *Deschampsia cespitosa* (a graminoid), in intra- or interspecific competition in a P deficient substrate amended with one of three forms of P and a mixture of all three. We tested the hypotheses that (1) *V. vitis-idaea* and *D. cespitosa* have preference for P forms leading to partitioning of soil P resources; (2) the ability to utilize different P forms is regulated by production of extracellular P-degrading enzymes; and (3) when plants are grown in interspecific competition, they acquire more P when a mixture of P forms are supplied, compared with single forms of P.

## Materials and Methods

### Growth substrate

The substrate mix was devised to ensure low background P availability and low pH, yet also to provide a degree of ecological relevance in terms of soil physical properties and microbial communities. Pots (6.5 cm diameter × 7 cm height) were filled with 36 g acid-washed sand, 2 g vermiculite, and 2 g air-dry peat to give a final pH of 5.4. The peat was collected from the upper 20 cm of Red Moss National Nature Reserve, Aberdeenshire (57.23ºN, −2.13ºW). The reserve is managed by the Scottish Wildlife Trust, and the most abundant plants species are *Calluna vulgaris* L. (Hull), *Eriophorum vaginatum* L., *Eriophorum angustifolium* Honck., *Erica tetralix* L., *Myrica gale* L., *Ulex europaeus* L., *Juncus effusus* L., *Betula pendula* Roth., *Carex* spp., *Sphagnum* spp., *Hypnum jutlandicum* Holmen & E.Warncke, and *Polytrichum commune* Hedw. (Hulme 2006). The peat samples were homogenized, and all the roots, plant residue, and litter together with undecomposed plant materials were removed. The peat was air-dried, sieved (2 mm), and stored at 5°C prior to use in the experiments. Seeds of *D. cespitosa* (L.) and *V. vitis-idaea* (L.) were obtained from Emorsgate Seeds Ltd and Chiltern Seeds Ltd. The seeds were surface sterilized with calcium hypochlorite for 20 min and washed with sterile water. The seeds were germinated on water agar for 4–6 days in the laboratory and transferred to the pots after 14 days.

### Experimental design

To study the ability of plants to use either simple inorganic P or complex organic P, five treatments were established; no P addition (control), sodium phosphate (SP; a simple inorganic P form), D-glucose 6 phosphate (DG6P; a simple organic P form), phytic acid sodium salt (PASS; a complex organic P form), and a mix of SP, DG6P, and PASS (MIX). The pots in all the treatments received the same amount of P (28.2 mg P·g^−1^ dwt). This was in addition to the background P that was already in the sand, vermiculite and peat growth medium (14.1 mg P·g^−1^ dwt), yielding a total of 1.69 g P for each pot. One day prior to planting, all the P treatments were added in powder form and mixed together with the growth substrate. P amendments were made only once.

Each pot received two plants, either as heterospecifics or conspecifics. Additional macro- and micronutrients were added to each pot with a solution consisting of 4 mmol·L^−1^ KNO_3_, 4 mmol·L^−1^ Ca(NO_3_)_2_, 1.5 mmol·L^−1^ MgSO_4_, 0.05 mmol·L^−1^ EDTA FeNa, 0.001 mmol·L^−1^ MnSO_4_, 0.001 mmol·L^−1^ ZnSO_4_, 0.001 mmol·L^−1^ CuSO_4_, 0.05 mmol·L^−1^ H_3_BO_3_, 0.0005 mmol·L^−1^ Na_2_MoO_4_, 0.1 mmol·L^−1^ NaCl, and 0.0002 mmol·L^−1^ Co(NO_3_)_2_ to ensure all the plants received required nutrients at sufficient level for optimal growth. Subsequently, all pots were watered with 8 mL distilled H_2_O every day. All pots were arranged randomly in five 5 blocks, and five replicates were used for each treatment, which comprised three combinations of plant treatments (*D. cespitosa* × *D. cespitosa*, *V. vitis-idaea* × *V. vitis-idaea* and *D. cespitosa* × *V. vitis-idaea*) and five P treatments (control, three individual P forms and a mix of all three) to give a total of 75 pots (five replicates × three plant treatments × five P treatments).

The experiment was conducted in a growth chamber for 8 weeks, starting at the middle of November 2011 until the middle of January 2012. Temperature in the growth chamber was 20 ± 1°C during daytime and 15 ± 1°C at night, and the light was set for 16 h daylight and 8 h night. After 8 weeks, all the plants were harvested. About 3–5 g soil was removed with 0.5 g used for assays of phosphomonoesterase and phytase activities. The remaining soil was dried for 48 h at 80°C. Soil adhering to plant roots was removed using tap water. Shoot and root parts were separated, and all the shoot samples were dried for at least 48 h at 80°C before weighing. Some of root samples were cut into small pieces (ca. 2 cm) for immediate assay of surface phosphomonoesterase and phytase activities.

### Assay of phosphomonoesterase and phytase activity

To understand the mechanism regulating the differences observed in plant biomass and nutrition, we measured phosphomonoesterase and phytase enzyme activity on *V. vitis-idaea* and *D. cespitosa* roots. Phosphomonoesterase activity was quantified using the substrate p-nitrophenyl phosphate (p-NPP), which breaks down to produce p-nitrophenol (p-NP), which is measured spectrophotometrically (Tabatabai and Bremner 1969). Phytase activity was quantified using the substrate sodium phytate and the enzyme activity measured according to the concentration of PO_4_ released (Li et al. 1997). The subsamples of fresh roots were cleaned in distilled water with a soft brush and assayed in 10-mL glass jars containing 4 mL pNPP in citrate buffer solution at pH 5.4 (the pH of the growth medium). The samples were placed in an incubator shaker for 30 min at 37°C after which 0.5 mL of supernatant was collected and added to 2 mL of 2 mol·L^−1^ NaOH to stop the reaction. The absorbance of samples was determined at 405 nm immediately after the reaction was stopped and compared with a standard curve of pNP.

For phytase activity, the samples were added to 0.1 mL sodium phytate in 0.4 mL acetate buffer at pH 5.4. The samples were placed in a shaking incubator for 30 min at 37°C, and the reaction was terminated by addition of 1 mL 10% or 0.4 mmol·L^−1^ trichloroacetic acid (TCA). Protein precipitated by TCA was removed by centrifugation at 10,000 *g* for 10 min, and the supernatant was analyzed for liberated PO_4_ using the ascorbic acid/molybdate method (Watanabe and Olsen 1965). After both assays, the fresh weight of all the root samples was determined, and enzyme activity expressed per unit fresh weight. The same procedure was followed for the assay of phosphomonoesterase and phytase activity in 0.5 g samples of soil.

### Measurement of N and P in shoots and soil

Samples were digested at 370°C for 4 h in a salicylic acid/sulfuric acid mix with a CuSO_4_/LiSO_4_ catalyst (Bremner and Mulvaney 1982) and diluted with distilled water (1:10). Determination of N and P was undertaken colorimetrically by auto-analysis (TecatorFIAStar 5010; Foss UK Ltd, Didcot, Oxfordshire, UK).

### Data analysis

The effect of P sources, plant species, competition, and the interactions between them were tested by general linear model in Minitab 16 using “pot” as random factor. A Tukey post hoc multiple comparison test was used to determine differences among individual mean values. Where necessary, data were normalized by either logarithmic or square-root transformation. The shoot biomass data for each of the three P sources and for each plant species were used to generate predicted shoot biomass values in response to a mix of all three P sources, assuming equal uptake of each component P source. We undertook a stratified bootstrapping procedure in R to generate the distribution of the predicted and observed values for the mix and their difference. We generated the distribution of observed means using 100,000 bootstrap samples for each individual P source and for the mix. At each iteration, we computed the difference between the mean for the mix (“observed”) and the mean of the means for the 3 P sources (“predicted”). A 95% confidence interval for the distribution of the differences not including zero indicated a significant difference between the observed and the predicted shoot biomass.

## Results

### Overall effects of P additions on shoot biomass

**Figure 1:**
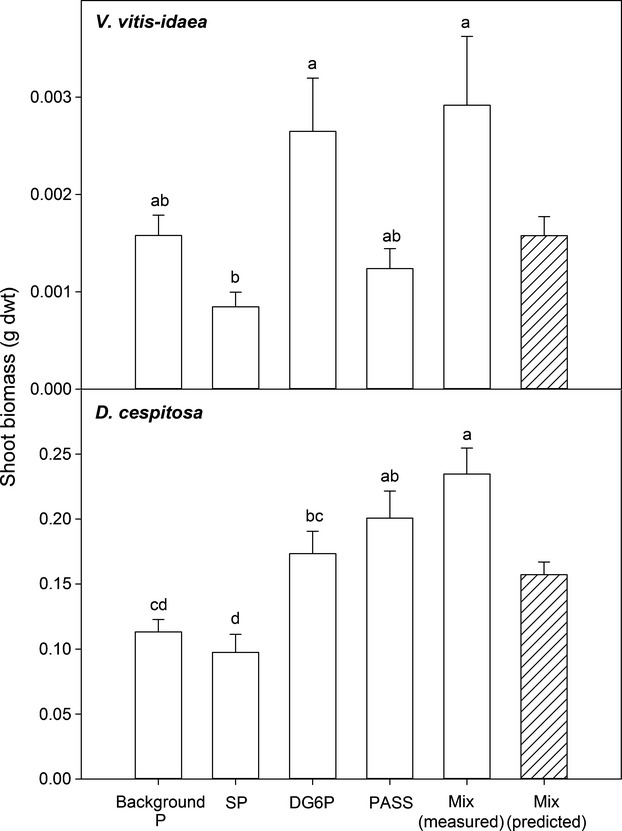
Mean (+SEM) shoot biomass of *Vaccinium vitis-idaea* and *Deschampsia cespitosa* in response to zero additions of P (background P) and addition of sodium phosphate (SP), D-glucose 6 phosphate (DG6P), phytic acid sodium salt (PASS), and a mixture of SP, DG6P, and PASS (Mix measured). Mix (predicted) is the mean biomass from the three single P form treatments. Bars sharing a letter are not significantly different (*P* > 0.05).

Generally, shoot biomass of *D. cespitosa* was stimulated by P additions, while the treatments had few significant effects on *V. vitis-idaea* shoot biomass, although overall the biomass responses were dependent on the type of P supplied (Fig. 1). These contrasting responses were reflected by a significant plant species × P treatment interaction (*P* < 0.001; F_4,149_ = 9.71). Shoot biomass of *V. vitis-idaea* in control treatments did not differ significantly compared with any P addition treatment; however, following additions of glucose phosphate (DG6P), the shoot biomass was 2.6 mg dwt, and in the MIX treatment, the shoot biomass was 2.7 mg dwt, representing an increase of around 75% relative to controls. There was no effect of additions of SP on the shoot biomass of either *V. vitis-idaea* or *D. cespitosa* (Fig. 1); in fact, the biomass of *V. vitis-idaea* was slightly less (but not significantly) in response to SP compared with controls and significantly less than the biomass in response to D6GP and MIX. The shoot biomass of *D. cespitosa* tended to increase alongside the complexity of P forms. In the MIX treatment, the shoot biomass was twice that of the controls, and in response to PASS, the shoots were 81% greater than controls in response to DG6P; both these differences were significant (Fig. 1). While not significantly different, the shoot biomass in response to D6GP was about 50% greater than the controls. The biomass produced by each species in response to MIX did not reflect the predicted responses, which were calculated based on the response to the P forms individually. In both species, the observed responses to MIX were the same or slightly greater than the maximum biomass produced in individual P treatments. For both *V. vitis-idaea* and *D. cespitosa*, the predicted responses were significantly less than the measured responses (difference with 95% confidence intervals: 0.00134 [0.00069; 0.0029] g dwt for *V. vitis-idaea* and 0.077 [0.036; 0.120] g dwt for *D. cespitosa*; Fig. 1).

### Effect of intra- and interspecific competition, and its interaction with P supply on growth and nutrition of plants

**Figure 2:**
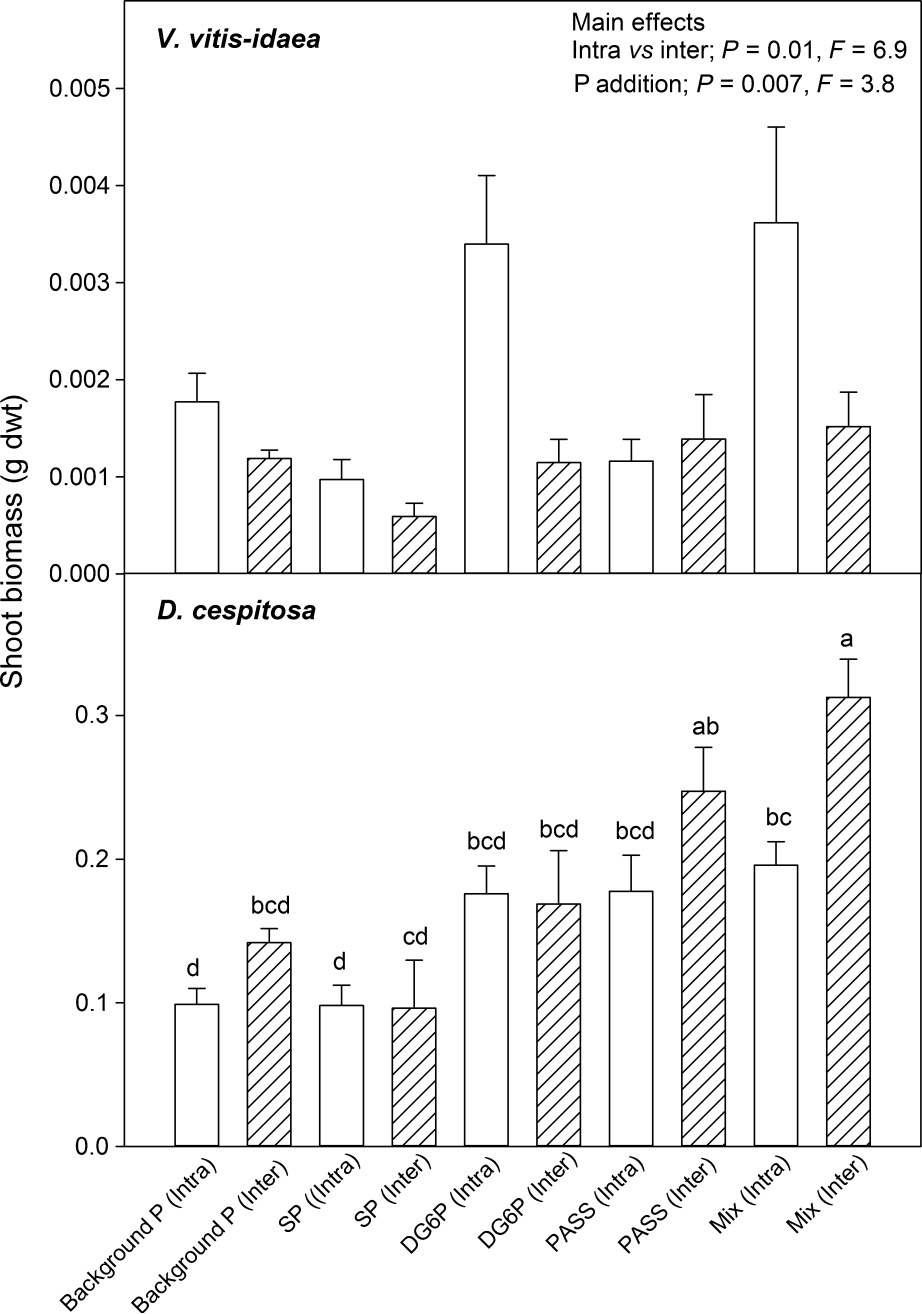
Mean (+SEM) above-ground biomass of *Vaccinium vitis-idaea* and *Deschampsia cespitosa* in response to P additions when grown in intra- (open) or interspecific (hatched) competition (see Fig. 1 for details of P treatments). Bars sharing a letter are not significantly different (*P* > 0.05). There was no significant P form × competition type interaction for *V. vitis-idaea*.

*Vaccinium vitis-idaea* produced two orders of magnitude less biomass than *D. cespitosa* (Fig. 2). There was a significant (F_1,74_ = 6.8; *P* = 0.01) overall effect of competition on shoot biomass of *V. vitis-idaea* but no interaction with P form. When analyzed across all P treatments, plants grown in competition with *D. cespitosa* produced 46% less biomass than when grown with conspecifics. PASS was the only P treatment where there was no measurable difference in shoot biomass of *V. vitis-idaea* when grown under intra- or interspecific competition. There was a significant interaction between P addition and competition type (F_4,74_ = 2.5; *P* = 0.049) on the biomass of *D. cespitosa* shoots (Fig. 2). The biomass response to P supply and competition was largely opposite to those observed for *V. vitis-idaea*. In controls, PASS and MIX, *D. cespitosa* tended to produce more biomass when grown with *V. vitis-idaea* compared with conspecifics, although this was only significant in the MIX treatment. In contrast, the responses of *D. cespitosa* in interspecific competition to DG6P and SP were neutral.

**Figure 3:**
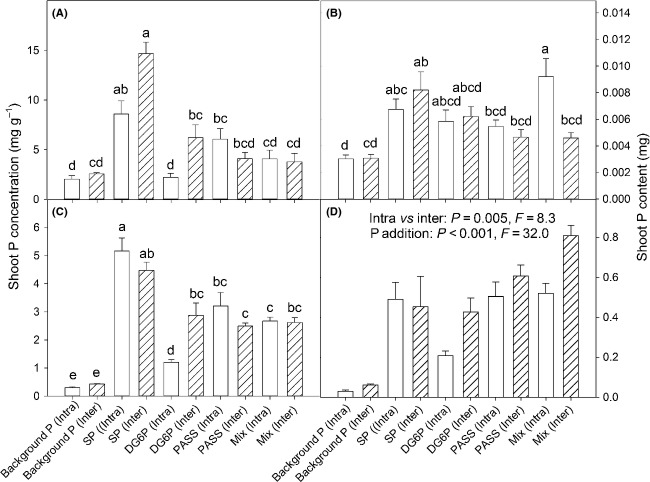
Mean (+SEM) shoot P concentration (A) and content (B) in *Vaccinium vitis-idaea*, and shoot P concentration (C) and content (D) in *Deschampsia cespitosa* in response to P additions when grown in intra- (open) or interspecific (hatched) competition (See Fig. 1 for details of P treatments). Within a panel, bars sharing a letter are not significantly different (*P* > 0.05). There was no significant P addition × competition interaction on shoot P content of *D. cespitosa*.

The concentration of P in shoots of both *V. vitis-idaea* and *D. cespitosa* was dependent on significant interactions between competition and P addition (for *V. vitis-idaea*: F_4,74_ = 4.4; *P* = 0.003. For *D. cespitosa:* F_4,74_ = 6.3; *P* < 0.001; Fig. 3). *V. vitis-idaea* had overall greater shoot P concentrations when supplied with SP, but these tended to be elevated when grown in interspecific competition. An even stronger significant effect of competition was seen in response to DG6P; here, plants had 2.5-times greater concentrations when grown with *D. cespitosa* compared with conspecifics. The concentration of P in *D. cespitosa* shoots that were provided with no additional P was around 0.5 mg·g^−1^, which was significantly less compared with all other treatments. Addition of SP resulted in plant shoots with approximately 20-times greater shoot P concentrations than the controls, although there was no effect of intra- versus interspecific competition in this treatment. In contrast, when DG6P was provided, shoot P concentrations significantly increased from 1.2 mg·g^−1^ in intraspecific competition to 2.9 mg·g^−1^ when grown with *V. vitis-idaea*; both concentrations being significantly greater than controls. When supplied with a mixture of P forms, there was no effect of competition on shoot P concentrations of both *D. cespitosa* and *V. vitis-idaea*.

The quantity of P in *V. vitis-idaea* shoots was affected by a significant interaction between P addition and competition (F_4,74_ = 3.1; *P* = 0.021; Fig. 3). Compared with controls, the amount of P was significantly greater when plants were grown in interspecific competition and supplied with SP, and when grown in intraspecific competition when supplied with a mixture of P forms. For *D. cespitosa*, there were only significant main effects of P addition and competition type. Compared with controls, plant shoots contained significantly more P regardless of P addition, while plants grown in interspecific competition also contained more P than when grown with conspecifics.

**Figure 4:**
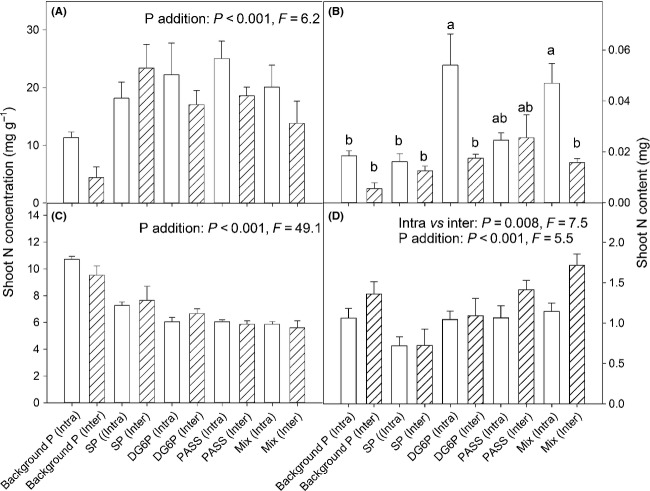
Mean (+SEM) shoot N concentration (A) and content (B) in *Vaccinium vitis-idaea*, and shoot N concentration (C) and content (D) in *Deschampsia cespitosa* in response to P additions when grown in intra- (open) or interspecific (hatched) competition (See Fig. 1 for details of P treatments). There was a significant P addition × competition interaction only for shoot N content of *V. vitis-idaea*. Within a panel, bars sharing a letter are not significantly different (*P* > 0.05).

The concentration of N in *D. cespitosa* and *V. vitis-idaea* tissues also differed after addition of various P forms (Fig. 4). In general, N concentrations in *V. vitis-idaea* shoots approximately doubled in response to P additions, although these effects were only significantly different from controls in SP, D6GP, and PASS. *D. cespitosa* shoots had significantly (F_4,74_ = 41.4; *P* < 0.001) smaller concentrations of N when they were supplied with P, regardless of the form. For example, in the MIX treatment, the concentration of N was nearly half that of the controls. The total amount of N in *V. vitis-idaea* shoots was dependent on a significant interaction between P addition and competition (F_4,71_ = 3.1; *P* = 0.050). This effect was largely driven by plants grown in intraspecific competition having substantially more N in shoots when supplied with D6GP and a mixture of P forms compared with controls. Less distinct effects were seen in the amount of N in *D. cespitosa* shoots, although plants in interspecific competition tended to contain more N than when grown with conspecifics. In addition, N content was significantly (F_4,74_ = 5.5; *P* < 0.001) greater in control plants and plants supplied with PASS and a mixture of P forms compared with those supplied with only SP.

### Activity of root surface enzymes and indices of competitiveness

**Figure 5:**
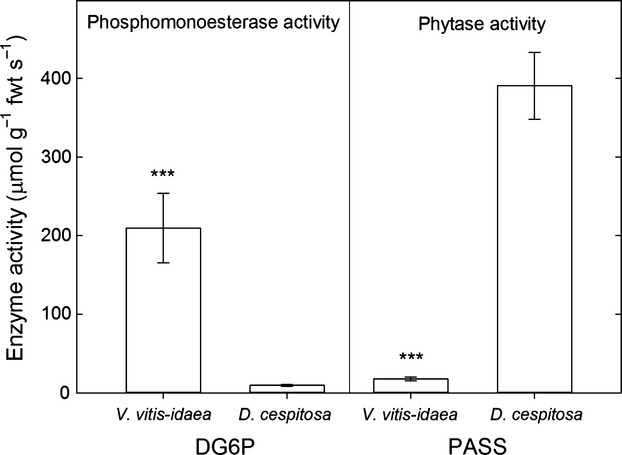
Mean (±SEM) root surface phosphomonoesterase and phytase activity of *Vaccinium vitis-idaea* and *D. cespitosa* in response to additions of D-glucose 6 phosphate (DG6P) and sodium phytate (PASS). Asterisks indicate significant (*P* < 0.001) differences between plant species within each P form.

Phytase activity on the root surface of *D. cespitosa* was 22-fold higher (T = 8.32; df = 18; *P* < 0.001) than *V. vitis-idaea* when grown with PASS (Fig. 5). In contrast, phosphomonoesterase activity in response to DG6P was significantly (*T* = −4.31; df = 18, *P* < 0.001) greater (approximately 200 *μ*mol·g·fwt^−1^·s^−1^) on the root surface of *V. vitis-idaea* than *D. cespitosa* (approximately 8.5 *μ*mol·g·fwt^−1^·s^−1^). These results indicate that *D. cespitosa* is more effective at using refractory organic P as a P source, and that *V. vitis-idaea* preferentially uses the simple labile organic form, DG6P.

## Discussion

We provide the first empirical test of partitioning for soil P by two plant species commonly found in P deficient ombrotrophic peatlands. In agreement with our first hypothesis and with the model proposed by Turner (2008), we found that the growth of both *V. vitis-idaea* and *D. cespitosa* responded differently depending on the form of P supplied to the growth medium. *D. cespitosa*, which is a relatively fast growing graminoid compared with *V. vitis-idaea*, was able to effectively use the organic P compound phytic acid (PASS), which is the least biological available of those used in the experiment. There was a tendency for *V. vitis-idaea* to produce the greatest shoot biomass in response to additions of DG6P. *D. cespitosa* seemed to acquire P from the stabilized and strongly sorbed PASS through the synthesis of phytase enzymes released from its root system, whereas production of phosphomonoesterases was negligible (Fig. 5). The production of solubilizing agents such as organic acids from *D. cespitosa* root exudates may also help promote phytase activity. For example, the combination of phytase and organic acids has been found to interact to affect plant utilization of inositol phosphate (Hayes et al. 2000; George et al. 2004). In contrast to *D. cespitosa*, the slower growing *V. vitis-idaea* had significantly more phosphomonoesterase on its root surfaces and produced virtually no phytase, that may have facilitated access the simple phosphate monoester, DG6P. DG6P is weakly sorbed and considered among the most abundant and available of organic P forms in soil (Condron et al. 2005). These data therefore suggest that P partitioning by these plants is regulated, at least in part, by production of root surface phosphatase enzymes and provides support for our second hypothesis. Thus, gaining a better understanding of the factors that regulate production, release, and activity of these enzymes is crucial for understanding plant community composition and its interactions with biogeochemical cycling. The findings corroborate past work showing that root surface enzymes contribute substantially to plant nutrition (Kroehler and Linkins 1988). One particularly important factor in regulating root surface P-solubilizing enzymes is deposition of atmospheric reactive N, which has been shown to stimulate enzyme activity to different extents depending on species identity (Johnson et al. 1999; Phoenix et al. 2004; Phuyal et al. 2007) and functionality (i.e., whether they are able to fix atmospheric N; Venterink 2011).

One further explanation of the contrasting activities of phosphatase enzymes found in the current experiment might be the different types of mycorrhizal fungi supported by the plants used; *V. vitis-idaea* forms ericoid mycorrhizas, and *D. cespitosa* forms arbuscular mycorrhizas (Harley and Harley 1987). The role of arbuscular mycorrhizal fungi in acquiring P via production of extracellular phosphatases is uncertain. One study estimated that utilization of P from phytic acid and subsequent translocation of P to *Trifolium repens* by external mycelium in a calcareous soil only contributed to around 3% of plant P nutrition (Feng et al. 2003). Our results from *V. vitis-idaea* generally support past work, which showed ericoid mycorrhizal fungi can utilize organic P sources by releasing a suite of phosphatases (although thus far phytase has yet to be tested), and could transport P back to host plants (Leake and Miles 1996; Myers and Leake 1996). The plant community found at our study site comprises a number of species with other root adaptations that enable them to acquire P efficiently, notably *M. gale* (cluster roots) and *Carex* spp. (dauciform roots; Playsted et al. 2006). However, these adaptations usually are nonspecific in terms of P acquisition. Instead, they enable plants to release organic acids and mobilize P in the rhizosphere, which can have significant positive effects on the growth and nutrition of neighboring co-occurring species without such adaptations (Johnson et al. 2004).

We found that the performance (biomass) of plants differed when supplied with a mixture of P forms compared with what would be predicted based on the assumption of equal utilization of P from each compound when applied separately. For *V. vitis-idaea*, the biomass when given a mix of three different P compounds was similar to the biomass produced in response to additions of DG6P only, that is, very positive. This suggests that the modest amount of P supplied in DG6P in the mixture (which was one-third of that applied when this compound was added on its own) was enough to satisfy plant P demand to the extent that other P forms in the mixture were not utilized. This finding is important because it provides evidence of an active foraging strategy whereby apparently more mobile forms of P (e.g., SP) are ignored in preference for DG6P. The response of *D. cespitosa* to mixed P sources differed to that of *V. vitis-idaea* in that it produced more biomass when given a mixture of P sources compared with all other single additions. This suggests that this species has preferences for P sources, as indicated in the model proposed by Turner (2008), rather than absolute specificity, as appears to be the case for *V. vitis-idaea*. In nature, plants are exposed to a vast diversity of P forms simultaneously, and so the approach we took in this experiment, where this “choice” was to a certain extent simulated gives confidence in our conclusion that resource partitioning for P likely occurs under field conditions. Nevertheless, it is important to also consider that the availability of the P forms, and not just their chemical composition, are likely to have differed in the experiment. It is also a possibility that the addition of one P form could have affected the availability of another when they were supplied in mixture. Further work is required to determine the relative importance of P availability and P chemical composition in driving competitive interactions in plant communities.

We hypothesized that competition between species would be dependent on the forms of P supplied to plants, and this hypothesis was partially supported by the results. The experimental design and duration were such that competition for above-ground resources (light) was unlikely to be a factor in determining competitive outcomes. If P supply had no role in mediating competitive outcomes, we would expect patterns in, for example, biomass to be similar under all P addition treatments. Yet, this was not the case; for example, with no added P, *D. cespitosa* tended to produce more biomass and acquire more N when grown with *V. vitis-idaea* compared when grown with a conspecific, while the reverse was the case for *V. vitis-idaea*. When provided with a mix of P sources, *D. cespitosa* produced significantly more biomass when grown with *V. vitis-idaea* than a conspecific. When supplied with PASS, *V. vitis-idaea* produced the same amount of biomass and acquired similar amounts of N when grown with conspecifics compared with heterospecifics. The ability of *V. vitis-idaea* to acquire P from DG6P, and the apparent poor ability of *D. cespitosa* to use this compound as a source of P resulted in the biomass of *D. cespitosa* being equal when grown with conspecifics or heterospecifics. Although *V. vitis-idaea* biomass and N acquisition remained less when grown with *D. cespitosa* compared with conspecifics under this P supply treatment, the competitive effect was lowest and competitive response the greatest in this treatment. It is of further interest that when given mixtures of P, the effects of competition were as predicted, with *D. cespitosa* producing more biomass and acquiring more N when grown with *V. vitis-idaea* and *vice versa*. Collectively, these findings suggest that while resource partitioning for P has a role in mediating competitive outcomes, other factors remain important. We now need to test whether preferential utilization of P forms regulates competitive outcomes in a greater range of species.

Although N was kept constant in the treatments, it was clear that the ability of plants to acquire it was dependent on species identity, competition, and P supply. For *V. vitis-idaea*, the N concentration in shoots tended to be greater in treatments receiving P, whereas the N concentration of *D. cespitosa* shoots was significantly reduced by P additions, indicating contrasting responses to the stoichiometry of soil mineral nutrient status. This result suggests that the interplay between the availability and mechanisms of uptake of N and P is likely to be critical for determining the relative contributions of these two species to plant community structure.

This study provides evidence of the ability of two peatland plant species to use different forms of soil P, thus supporting the hypothesis that resource partitioning for soil P is an important process. Although this is the first direct evidence of resource partitioning of soil P, how these findings can be applied to natural communities and their management requires more work. Firstly, we used just two plant species, whereas most natural peatland communities have a greater number of species representing broad functional and taxonomic groups (Hulme 2006). Greater species diversity could be reflected by even wider abilities to access and utilize P forms in peatlands. Secondly, we used only three P forms (plus the mix), which may not represent fully the complex P pool in many ecosystems. In peatland, P storage can range between 0.2 and 0.5 g·m^−2^ (Whigham et al. 2002) and comprises numerous organic P forms including inositol phosphate, orthophosphate diester, pyrophosphate, phosphonates (Turner et al. 2004), phospholipids, nucleotides, sugar phosphate (Tisdale et al. 1985) phytates, nucleic acids, phosphate ester, and adenosine phosphates (Jayachandran et al. 1992; Marschner 1995). Finally, we grew plants for just 8 weeks; therefore, in this study, we only focussed on the early stages of below-ground competition between *D. cespitosa* and *V. vitis-idaea*, and recent evidence suggests growth and nutrient capture by competing plants is highly dynamic throughout their life span (Trinder et al. 2012). Despite these uncertainties, it is clear that resource partitioning of soil inorganic and organic P is a crucial but understudied process that can have profound effects on plant productivity, growth, and competition.

## References

[b1] Antibus RK, Sinsabaugh RL, Linkins AE (1992). Phosphatase activities and phosphorus uptake from inositol phosphate by ectomycorrhizal fungi. Can. J. Bot.

[b2] Bremner JM, Mulvaney CS, Page AC, Miller RH, Keeney DR (1982). Salicylic acid-thiosulphate modification of Kjeldahl method to include nitrate and nitrite. Agronomy 9: methods of soil analyses. Part 2. Chemical and microbiological properties.

[b3] Condron LM, Turner BL, Cade-Menun BJ, Sims JT, Sharpley AN (2005). The chemistry and dynamics of soil organic phosphorus. Phosphorus: agriculture and the environment.

[b4] Criquet S, Ferre E, Farnet AM, Petit LJ (2004). Annual dynamics of phosphatase activities in an evergreen oak litter: influence of biotic and abiotic factors. Soil Biol. Biochem.

[b5] Cross AF, Schlesinger WH (2001). Biological and geochemical controls on phosphorus fractions in semiarid soils. Biogeochemistry.

[b6] Felipe MR, Pozuelo JM, Cintas AM (1979). Acid phosphatase localization at the surface of young corn roots. Agrochemica.

[b7] Feng G, Song YC, Li XL, Christie P (2003). Contribution of arbuscular mycorrhizal fungi to utilization of organic sources of phosphorus by red clover in calcareous soil. Appl. Soil Ecol.

[b100] Fransson AM, Van Aarle IM, Olsson PA, Tyler G (2003). *Plantago lanceolata* L. and *Rumex acetosella* L. differ in their utilisation of soil phosphorus fractions. Plant Soil.

[b8] George TS, Richardson AE, Hadobas PA, Simpson RJ (2004). Characterization of transgenic *Trifolium subterraneum* L. which expresses phy A and release extracellular phytase: growth and P nutrition in laboratory media and soil. Plant, Cell Environ.

[b9] Harley JL, Harley EL (1987). A check-list of mycorrhiza in the British flora. New Phytol.

[b10] Harrison AF (1987). Soil organic phosphorus: a review of world literature.

[b11] Hayes JE, Richardson AE, Simpson RJ (2000). Components of organic phosphorus in soil extracts that are hydrolysed by phytase and acid phosphatase. Biol. Fertil. Soils.

[b12] Hulme PD (2006).

[b13] Jayachandran K, Schwab AP, Hetrick BAD (1992). Mineralization of organic phosphorus by vesicular-arbuscular mycorrhizal fungi. Soil Biol. Biochem.

[b14] Johnson D, Leake JR, Lee JA (1999). The effects of quantity and duration of simulated pollutant nitrogen deposition on root surface phosphatase activities in calcareous and acid grasslands: a bioassay approach. New Phytol.

[b15] Johnson D, Vandenkoornhuyse PJ, Leake JR, Gilbert LA, Booth RE, Grime JP (2004). Plant communities affect arbuscular mycorrhizal fungal diversity and community composition in grassland microcosms. New Phytol.

[b16] Kroehler CJ, Linkins AE (1988). The root surface phosphatase of *Eriophorum vaginatum*: effects of temperature, pH, substrate concentration and inorganic phosphorus. Plant Soil.

[b17] Leake JR, Miles W (1996). Phosphodiesters as mycorrhizal P sources. I. Phosphodiesterase production and utilization of DNA as a phosphorus source by the ericoid mycorrhizal fungus *Hymenoscyphusericae*. New Phytol.

[b18] Li M, Osaki M, Rao IM, Tadano T (1997). Secretion of phytase from the roots of several plant species under phosphorus-deficient conditions. Plant Soil.

[b19] Macklon AES, Grayston SJ, Shand CA, Sim A, Sellars S, Ord BG (1997). Uptake and transport of phosphorus by *Agrostis capillaris* seedlings from rapidly hydrolysed organic sources extracted from ^32^P-labelled bacterial cultures. Plant Soil.

[b20] Marschner H (1995). Mineral nutrition of higher plants.

[b21] McKane RB, Johnson LC, Shaver GR, Nadelhoffer KJ, Rastetter EB, Fry B (2002). Resource-based niches provides a basis for plant species diversity and dominance in arctic tundra. Nature.

[b22] Myers MD, Leake JR (1996). Phosphodiesters as mycorrhizal P sources. II. Ericoid mycorrhiza and the utilization of nuclei as a phosphorus and nitrogen sources by *Vaccinium macrocarpon*. New Phytol.

[b23] Phoenix GK, Booth RE, Leake JR, Read DJ, Grime JP, Lee JA (2004). Simulated pollutant nitrogen deposition increases P demand and enhances root-surface phosphatase activities three plant functional types in a calcareous grassland. New Phytol.

[b24] Phuyal M, Artz RRE, Sheppard L, Leith I, Johnson D (2007). Long-term nitrogen deposition increases phosphorus limitation of bryophytes in an ombrotrophic bog. Plant Ecol.

[b25] Playsted CWS, Johnston ME, Ramage CM, Edwards DG, Cawthray GR, Lambers H (2006). Functional significance of dauciform roots: exudation of carboxylates and acid phosphatase under phosphorus deficiency in *Caustis blakei* (Cyperaceae). New Phytol.

[b26] Sahu MK, Sivakumar K, Kannan L (2007). Phosphate solubilising actinomycetes in the estuarine environment: an inventory. J. Environ. Biol.

[b27] Tabatabai MA, Bremner JM (1969). Use of p-nitrophenylphosphate for assay of soil phosphatase activity. Soil Biol. Biochem.

[b28] Tarafdar JC, Claassen N (2003). Organic phosphorus utilization by wheat plants under sterile conditions. Biol. Fertil. Soils.

[b29] Tisdale SL, Nelson WL, Beaton JD (1985). Soil fertility and fertilizers.

[b30] Trinder C, Brooker R, Davidson H, Robinson D (2012). Dynamic trajectories of growth and nitrogen capture by competing plants. New Phytol.

[b31] Turner BL (2008). Resource partitioning for soil phosphorus: a hypothesis. J. Ecol.

[b32] Turner BL, McKelvie ID, Haygarth PM (2002a). Characterisation of water-extractable soil organic phosphorus by phosphatase hydrolysis. Soil Biol. Biochem.

[b33] Turner BL, Papházy MJ, Haygarth PM, McKelvie ID (2002b). Inositol phosphates in the environment. Philos. Trans. R. Soc. Lond. B. Biol. Sci.

[b34] Turner BL, Cade-Menun BJ, Westermann DT (2003a). Organic phosphorus composition and potential bioavailability in semi-arid arable soils of the western United States. Soil Sci. Soc. Am. J.

[b35] Turner BL, Mahieu N, Condron LM (2003b). Quantification of myo-inositol hexakisphosphate in alkaline soil extracts by solution ^31^P NMR spectroscopy and spectral deconvolution. Soil Sci.

[b36] Turner BL, Baxter R, Mahieu N, Sjögersten S, Whitton BA (2004). Phosphorus compounds in subarctic Fennoscandian soils at the mountain birch (*Betula pubescens*) – tundra ecotone. Soil Biol. Biochem.

[b37] Venterink HO (2011). Legumes have a higher root phosphatase activity than other forbs, particularly under low inorganic P and N supply. Plant Soil.

[b38] Wassen MJ, Venterink HO, Lapshina ED (2005). Endangered plants persist under phosphorus limitation. Nature.

[b39] Watanabe FS, Olsen SR (1965). Test of an ascorbic acid method to get phosphorus in water and NaHCO_3_, extracts from soil. Soil Sci. Soc. Am. J.

[b40] Whigham D, Pittek M, Hofmockel KH, Jordan T, Pepin AL (2002). Biomass and nutrient dynamics in restored wetlands on the outer coastal plain of Maryland, USA. Wetlands.

[b41] Wyss M, Brugger R, Kronenberger A, Remy R, Fimbeld R, Oesterhelt G (1999). Biochemical characterization of fungal phytase (my-inositol hexakisphosphatephosphohydrolases), catalytic properties. Appl. Environ. Microbiol.

